# Bacterial Community Influences the Effects of *Lactobacillus acidophilus* on Lipid Metabolism, Immune Response, and Antioxidant Capacity in Dogs

**DOI:** 10.3390/ani14091257

**Published:** 2024-04-23

**Authors:** Aipeng Mao, Xiaoli Chen, Weigang Zhao, Weixiao Nan, Yao Huang, Yalong Sun, Haihua Zhang, Chao Xu

**Affiliations:** 1 College of Animal Science and Technology, Jilin Agricultural University, Changchun 130118, China; mao443418199@163.com (A.M.);; 2 Institute of Special Animal and Plant Sciences, Chinese Academy of Agricultural Sciences, Changchun 130112, China; 3 Hebei Key Laboratory of Specialty Animal Germplasm Resources Exploration and Innovation, College of Animal Science and Technology, Hebei Normal University of Science and Technology, Qinhuangdao 066004, China

**Keywords:** *Lactobacillus acidophilus*, dog, serum indices, bacterial community, correlation analysis

## Abstract

**Simple Summary:**

In the process of domestication and urbanization, people and small companion animals share, to varying degrees, their dietary style and living environment, and they also encounter similar lifestyle challenges, such as obesity and other diseases. In our study, *Lactobacillus acidophilus* can improve the body’s lipid metabolism and immune and antioxidant capacity by regulating the relative abundance of intestinal bacterial community, which enables companion animals more easily adapt to the lifestyle of lower level of physical activity in the city, thereby improving animal health and well-being.

**Abstract:**

*Lactobacillus acidophilus* (*L. acidophilus*), the most prevalent probiotic, has demonstrated the ability to improve the relative abundance of intestinal microorganisms and boost immunity. However, the underlying mechanisms of these effects remain unclear. This study evaluated body weight, nutrient apparent digestibility, serum indices, and bacterial communities in Chinese rural dogs from a *L. acidophilus* supplementation group (*Lactobacillus acidophilus*, *n* = 6) and a control group (CON, *n* = 6). The results indicated that *L. acidophilus* had no significant impact on the body weight and apparent nutrient digestibility of Chinese rural dogs. In comparison with the CON group, *L. acidophilus* significantly reduced the levels of cholesterol (CHO) and increased the levels of IgA, IFN-α, and T-AOC. Bacterial diversity indices were significantly reduced in the LAC group compared to the CON groups, and MetaStat analysis demonstrated notable distinctions in 14 bacterial genera between the groups. These bacterial genera exhibited correlations with physiological indices such as CHO, IgA, IFN-α, and T-AOC. In conclusion, *L. acidophilus* can modulate lipid metabolism, immunity, and antioxidant capacity by regulating the relative abundance of specific bacterial communities, which helps dogs to adapt to today’s lifestyle.

## 1. Introduction

*Lactobacillus acidophilus* (*L. acidophilus*) is a prevalent probiotic characterized by good resistance against acid and bile salts [1], which ensures its survival in hostile environment. And it also can colonize complex gastrointestinal ecosystem, with beneficial effects accumulating over a longer period of time compared to non-colonizing probiotics [2]. Beyond its survival mechanisms, *L. acidophilus* exhibits the ability to metabolize dietary phytoglycosides and externalize its bioactive phytochemicals [3]. Furthermore, it plays a pivotal role in the body’s immune system, with studies demonstrating its effectiveness in reducing cholesterol (CHO) levels and combating pathogenic bacteria [4,5]. Its potential extends to improving the progression of nonalcoholic steatosis by lowering CHO, establishing it as an effective strategy for treating nonalcoholic fatty liver disease (NAFLD) [6]. And *L. acidophilus* also has demonstrated promising potential in ameliorating type 2 diabetes and obesity by modulating CHO levels and the abundance of the intestinal bacterial community in murine models [7,8].

In the process of domestication and urbanization, dogs and humans exhibit remarkable consistency in terms of dietary structure and living environment, particularly in developed countries [9,10,11]. There are also similarities between dog and human gut microbiota in terms of gene content and response to diet [12]. Given that most dogs are subjected to high-carbohydrate diets and encounter similar lifestyle challenges as humans [13], the metabolic dysfunctions associated with obesity in dogs can result in a significant increase in serum total CHO and triglycerides (TG) [14,15]. With dogs assuming pivotal roles as companions in people’s lives, the management of their nutrition and health are worthy of investigation and consideration. Notably, *L. acidophilus* has been reported to enhance growth in dogs [16]. Specifically, the *L. acidophilus* strain DSM13241 can be successfully incorporated into a dry diet and survive in the canine or feline gastrointestinal tract. This incorporation is associated with an elevation in the concentration of IgG in dogs, accompanied by an increase in the relative abundance of fecal Lactobacilli and a decrease in the relative abundance of Clostridial organisms [17], and it has been observed to reduce fecal pH, elevate the relative abundance of beneficial *Lactobacillus* and *L. acidophilus*, while decreasing the relative abundance of *Clostridium* spp. and *Enterococcus faecalis* in cats [18]. Additionally, *L acidophilus* D2/CSL (CECT 4529) significantly improved the nutritional status and fecal parameters in dogs [19]. These findings suggest that this *L. acidophilus* strain holds the potential to enhance the balance of the bacterial community in dogs and cats, thereby contributing to improved intestinal health and immune function.

This study investigated the effects of *L. acidophilus* on digestibility, metabolism, and serum indices in dogs, with the main aim of developing a novel probiotic to provide valuable insights into dog nutrition and health research, offering guidance for the formulation of pet food.

## 2. Materials and Methods

### 2.1. L. acidophilus

*L. acidophilus* was isolated and preserved in our laboratory. The DeMan-Rogosa-Sharpe (MRS) medium was used to resuscitate and passage cultures for 36 h at 37 °C, and the concentration of bacterial solution was determined to be 1 × 10^8^ CFU/mL.

### 2.2. Experimental Animals and Study Design

A total of 12 three-month-old Chinese rural dogs, with a mean body weight of 4.68 ± 0.93 kg, were recruited for this study, and the ratio of male to female dogs was equal. Each dog was housed in a cage, all animals were kept under controlled conditions of room temperature, humidity, and a 12/12 h light/dark cycle, and the dogs were provided with two daily feedings and unrestricted access to laboratory water. One month prior to the commencement of the experiment, all of the dogs underwent vaccination and deworming procedures following routine immunization protocols. Specifically, each animal received the canine quadruple vaccine and underwent deworming based on fecal examination and overall physical condition.

After a minimum acclimatization period of 3 days in laboratory conditions, the dogs were randomly assigned to either the control group (CON) or the *L. acidophilus* group (LAC) with 6 replicates per group. All of the animals were fed dry kibble food twice daily and had unrestricted access to laboratory water to meet the nutrient requirement of dogs at maintenance [20]. Dogs in the LAC group received 1 mL *L. acidophilus* culture solution, while those in the CON group were administered an equal volume (1 mL) of normal saline orally after feeding. After each feeding, the syringe containing the bacteria solution or normal saline was injected into the dog’s mouth to ensure full swallowing. The average daily feed intake per animal was approximately 200 g/d during the pretest, and the dose of probiotics was calculated to be 2 × 10^8^ CFU/day (European Standard No. EN 15878:2021; Animal feeding stuffs: Methods of sampling and analysis -Detection and enumeration of lactobacillus spp. used as feed additive. UNE EN 15787:2022; Animal feeding stuffs: Methods of sampling and analysis - Detection and enumeration of *Lactobacillus* spp. used as feed additive. UNE EN 15787:2022. compound feeds, meal or pellets which contain about 10^9^ CFU/kg *Lactobacillus* species) [19,21]. The entire experiment spanned 28 days, and the composition and nutritional content of the basal diet are detailed in Table 1.

### 2.3. Sample Collection

On the 20th day of the experiment, fecal samples from all dogs were collected over four consecutive days. The samples were fixed with 10% H_2_SO_4_ and stored at −20 °C for the subsequent digestibility trial. On the 28th day of the experiment, 10 mL of blood per dog was obtained from the dorsal subcutaneous cephalic vein of the forelimb of the animal using a disposable negative pressure sampling vessel with separation glue coagulant. The collected blood samples were then centrifuged at 3500× *g* for 10 min at 4 °C to extract the serum. The serum was utilized to determine the concentrations of serum indices immediately. Furthermore, approximately 5 g of feces from the animals were collected immediately after defecation at the conclusion of the experiment. These fecal samples were stored at −80 °C for subsequent bacterial community analysis.

### 2.4. Measurement of Body Weight

On the morning of the 7th, 14th, 21st, and 28th day of the experiment, the body weight of the experimental animals was measured on an empty stomach. The total weight gain (TWG), average daily weight gain (ADG), and weight gain rate (WGR) were calculated using the following formulas:

TWG = final weight − initial weight

ADG = (final weight − initial weight)/28

WGR = (final weight − initial weight)/initial weight

### 2.5. Measurement of Nutrient Apparent Digestibility

The diet and fecal samples underwent thawing and drying at 65 °C for 48 h, followed by grinding through a 1 mm sieve before subsequent analysis. Duplicate analyses were conducted on both feed and fecal samples to determine dry matter (DM, method No. 934.01), crude protein (CP, method No. 954.01), and ether extract (EE, method No. 920.39), in accordance with AOAC methods [22]. Carbohydrate content was calculated using the formula: Carbohydrate (%) = DM − CP − EE − Ash (crude ash).

### 2.6. Measurement of Serum Indices

The levels of serum lipid indices were assessed using Biosino Bio-Technology and Science Inc. (Beijing, China) by an automatic biochemical analyzer (VITALIB-E). This included the measurement of triglycerides (TG), cholesterol (CHO), high-density lipoprotein cholesterol (HDL-C), and Low-Density Lipoprotein Cholesterol (LDL-C). The levels of immune indices (IgA, IgM, IFN-α, IL-2) were determined using Enzyme-Linked Immunosorbent Assay (ELISA) kits from Jianglai Biocompany, (Shanghai, China). Additionally, the levels of antioxidant indices (T-AOC, SOD, GSH-Px) were measured using commercial kits from Jiancheng Bioengineering Institute (Nanjing, China).

### 2.7. Bacterial Community Analysis

Total genomic DNA in the fecal samples was extracted using the SDS method, and the purity and concentration of the DNA were verified through 1% agarose gel electrophoresis. Specific primers with barcodes (341F: 5′-CCTAYGGGRBGCASCAG-3′, 806R: 5′-GGACTACNNGGGTATCTAAT-3′) were employed for amplification, depending on the selected region for sequencing [23]. The resulting amplicons were sequenced on the Illumina Novaseq 6000 (San Diego, CA, USA) to generate paired-end reads.

Species annotation was conducted using the QIIME2 software (Verision 1.9.1) for 16S rRNA gene sequences, with the Silva Database serving as the annotation database. GraphPad Prism 9.4.1 software was utilized to analyze differences in α diversity indices and bacterial composition between groups. To assess the complexity of bacterial community composition and compare differences among groups, β diversity was calculated based on Jaccard and unweighted UniFrac distances using QIIME2. Principal coordinate analysis (PCoA) was performed to generate principal coordinates and visualize sample differences in multidimensional data. The PCoA results were visualized using the ade4 and ggplot2 packages in R software (Version 2.15.3).

### 2.8. Statistical Analysis

Statistical analysis was conducted using GraphPad Prism 9.4.1 software. The *t* test was employed to assess the statistical significance of body weight, nutrient apparent digestibility, serum indices, and α diversity indices between the CON and LAC groups. Anoism analysis was utilized to calculate β diversity indices. The differentiation of bacterial genera between the two groups was analyzed using MetaStat. Statistical significance was defined as *p* < 0.05 (*), *p* < 0.01 (**), and *p* < 0.001 (***). The correlations between bacterial community and physiological indices were evaluated using the Spearman rank correlation coefficient. Network visualization was performed using R 4.2.2 to illustrate the relationships.

## 3. Results

### 3.1. Effects of Lactobacillus acidophilus on Body Weight in Chinese Rural Dogs

As indicated in Table 2, the body weight on days 7, 14, 21, and 28 exhibited a slight increase in the LAC group compared to the CON group; however, the differences were not statistically significant (*p* > 0.05). Similarly, the ADG and WGR also displayed a marginal increase, but the differences were not statistically significant (*p* > 0.05).

### 3.2. Effect of Lactobacillus acidophilus on Apparent Digestibility of Nutrients in Chinese Rural Dogs

As illustrated in Table 3, the apparent digestibility of nutrients, including DM, CP, EE, and carbohydrates, were not statistically significant compared to the CON group (*p* > 0.05).

### 3.3. Effect of Lactobacillus acidophilus on Serum Indices in Chinese Rural Dogs

**Figure 1:**
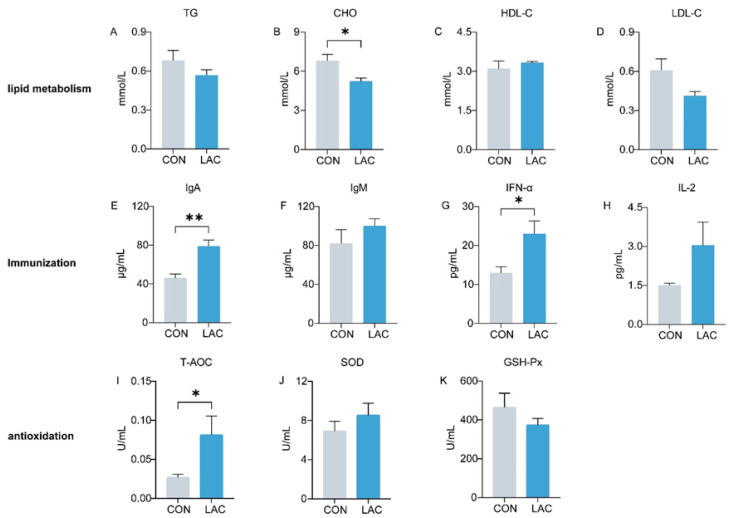
Effects of *L. acidophilus* on serum indices of Chinese rural dogs. (**A**–**D**) Comparison of lipid metabolism indices between the CON and LAC groups. (**E**–**H**) Comparison of immunological indices between the CON and LAC groups. (**I**–**K**) Comparison of antioxidant indices between the CON and LAC groups. * and ** indicate *p* < 0.05 and *p* < 0.01, respectively. TG: triglycerides, CHO: cholesterol, HDL-C: high-density lipoprotein cholesterol, LDL-C: low-density lipoprotein, IgA: immunoglobulin A, IgM: immunoglobulin M, IFN-α: interferon α, IL-2: interleukin 2, T-AOC: total antioxidant capacity, SOD: Superoxide dismutase, GSH-Px: Glutathione Peroxidase.

Figure 1 depicts the impact of *L. acidophilus* on serum lipid metabolism, immunity, and antioxidation in dogs. Comparative analysis with the CON group revealed a significant reduction in CHO levels in the LAC group (*p* < 0.05) (Figure 1B). TG and LDL exhibited a decrease in the LAC group, although the differences were not statistically significant (*p* > 0.05) (Figure 1A,D). In terms of immune indices, IgA in the LAC group demonstrated a remarkable increase compared to the CON group (*p* < 0.01) (Figure 1E), and IFN-α exhibited a significant elevation (*p* < 0.05) (Figure 1G). IgM and IL-2 also showed an increase, though the differences were not significant (*p* > 0.05) (Figure 1F,H). Regarding antioxidation, T-AOC showed a significant increase in the LAC group (*p* < 0.05) (Figure 1I), whereas the activity of SOD did not exhibit a significant difference between the two groups (*p* > 0.05) (Figure 1J).

### 3.4. Effects of Lactobacillus acidophilus on the Bacterial Community in Chinese Rural Dogs

**Figure 2:**
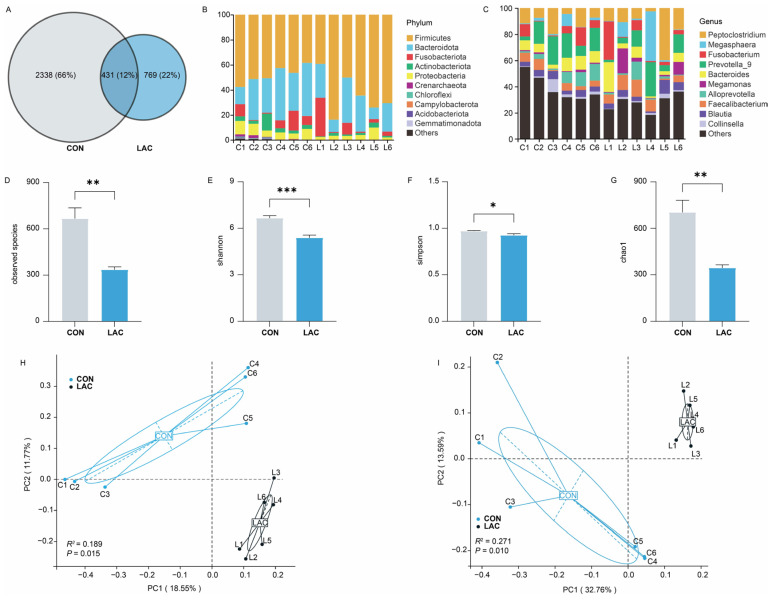
Effects of *L. acidophilus* on the composition and diversity of bacterial communities in Chinese rural dogs. (**A**) Shared and unique bacterial ASVs between the two groups. (**B**) Bacterial community composition at the phylum level in feces. (**C**) Bacterial community composition at the genus level in feces. (**D**) Composition of observed species of bacterial diversity between the two groups. (**E**) Composition of Shannon indices of bacterial diversity between the two groups. (**F**) Composition of Simpson indices of bacterial diversity between the two groups. (**G**) Composition of the Chao 1 indices of bacterial diversity between the two groups. (**H**) Composition of bacterial community based on Jaccard distance between the two groups. (**I**) Composition of bacterial community based on unweighted UniFrac distance between the two groups. *, ** and *** indicate *p* < 0.05, *p* < 0.01 and *p* < 0.001, respectively.

To explore the impact of *L. acidophilus* on the bacterial community of Chinese rural dogs, a total of 431 amplicon sequence variants (ASVs) were shared in the two groups, with 2338 and 769 unique ASVs in the CON and LAC groups, respectively (Figure 2A). At the phylum level, *Firmicutes* and *Bacteroidota* were the most abundant bacteria in both groups, followed by *Proteobacteria* in the CON group and *Fusobacteriota* in the LAC group, collectively accounting for 86.41% in the CON group and 93.99% in the LAC group of the bacterial composition (Figure 2B). Moreover, 476 bacterial genera were identified from the feces of dogs, with *Prevotella_9* (CON = 14.30%, LAC = 10.59%) and *Peptoclostridium* (CON = 10.88%, LAC = 14.57%) being the predominant genera (Figure 2C).

Assessment of bacterial community diversity between the CON and LAC groups revealed that the number of observed species, the Shannon and Chao 1 indices, were significantly lower in the LAC group than in the CON group (*p* < 0.01) (Figure 2D,E,G), while the Simpson indices were significantly lower in the LAC group than in the CON group (*p* < 0.05) (Figure 2F). This suggests that *L. acidophilus* significantly reduces the species number and bacterial community richness in feces. The PCoA results demonstrated significant differences in fecal bacterial community and its composition between the two groups based on Jaccard (Anoism: *R*^2^ = 0.189, *p* = 0.015) and unweighted UniFrac (Anoism: *R*^2^ = 0.271, *p* = 0.010) distances, explaining at least 30.32% and 46.35% of the variation, respectively (Figure 2H,I).

**Figure 3:**
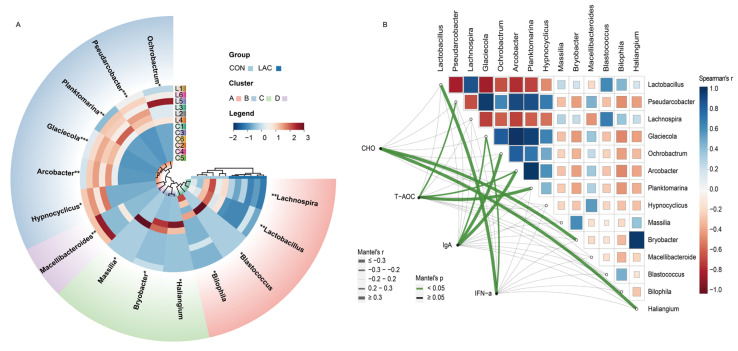
Correlation analysis between bacterial community differences and physiological indices in Chinese rural dogs. (**A**) Comparison of different bacterial genera by MetaStat analysis. (**B**) Analysis of correlation between bacterial genera and physiological indices. *, ** and *** indicate *p* < 0.05, *p* < 0.01 and *p* < 0.001, respectively. Spearman’s correlation coefficients are denoted with a color gradient. Physiological indices were related to each bacterial genus community composition environmental factor by Mantel tests. Edge width corresponds to the Mantel’s r statistic for the corresponding distance correlations, and edge color denotes the statistical significance.

Furthermore, we identified 14 differential genera between the two groups through MetaStat analysis (Figure 3A). Specifically, the relative abundance of *Pseudarcobacter* and *Glaciecola* was extremely significantly increased in the LAC group compared to the CON group (*p* < 0.001). *Planktomarina*, *Macellibacteroides*, and *Arcobacter* were also extremely significantly increased in the LAC group compared to the CON group (*p* < 0.01). *Ochrobactrum* and *Hypnocyclicus* were significantly increased in the LAC group compared to the CON group (*p* < 0.05). However, *Lactobacillus* and *Lachnospira* were significantly decreased in the LAC group compared to the CON group (*p* < 0.01), while *Massilia*, *Bryobacter*, *Haliangium*, *Bilophila*, and *Blastococcus* were significantly decreased in the LAC group compared to the CON group (*p* < 0.05) (Figure 3A).

For a deeper exploration of potential connections between the different bacterial community and physiological indices, Spearman’s rank correlation analysis was conducted to ascertain the correlation coefficients among differentially abundant bacteria. The results revealed that *Lactobacillus*, *Pseudarcobacter*, *Lachnospira*, *Glaciecola*, *Ochrobactrum*, *Arcobacter*, *Planktomarina*, and *Hypnocyclicus* exhibited the most significant correlations (Figure 3B). Correlation coefficients between differentially abundant bacteria and key indicators such as CHO, T-AOC, IgA, and IFN-α were analyzed by the Mantel test. The results indicated that *Bryobacter* and *Haliangium* were correlated with CHO, *Pseudarcobacter*, *Arcobacter*, and *Planktomarina* were correlated with T-AOC, *Glaciecola*, *Ochrobactrum*, and *Arcobacter* were correlated with IgA, while *Lactobacillus* and *Glaciecola* were correlated with IFN-α (Figure 3B).

## 4. Discussion

Probiotics, defined as living microorganisms conferring health benefits when administered in sufficient quantities, play a vital role in promoting host well-being [24]. Among these, *L. acidophilus* stands out as the most commonly utilized probiotic in various applications. Research has demonstrated its efficacy, such as reducing yolk cholesterol in laying hens and positively impacting their health and performance when incorporated into their diet [25]. *L. acidophilus* NCFM has also been found to influence intestinal carbohydrate, bile acid, and vitamin E metabolism [26]. The impact of probiotics on animal body weight is known to be contingent upon factors such as specific strains, probiotic dosage, and intervention duration [27]. However, our study revealed that *L. acidophilus* did not exert significant effects on body weight and the apparent digestibility of nutrients in Chinese rural dogs. This outcome could be attributed to the relatively short intervention duration of *L. acidophilus* and the heterogeneity of Chinese rural dogs, potentially obscuring any significant changes in animal body weight within the test results.

Hypercholesterolemia can result from various factors, including gain-of-function mutations in proprotein convertase subtilisin kexin 9 (PCSK9), leading to decreased low-density lipoprotein (LDL) receptor levels, and loss-of-function variants associated with reduced LDL-cholesterol (LDL-C) levels and decreased coronary heart disease (CHD) risk [28,29,30,31]. Studies indicate that *L. acidophilus* can contribute to lowering cholesterol (CHO) levels through mechanisms such as inhibiting 3-hydroxy-3-methylglutaryl CoA reductase and promoting the excretion of dietary cholesterol in feces through co-precipitation of deconjugated bile acids in the intestine and/or adsorption by organisms [2]. Furthermore, *L. acidophilus* NS1 has been shown to increase the expression of sterol regulatory element-binding protein 2 (SREBP2) and low-density lipoprotein receptor (LDLR) in the liver, resulting in decreased total cholesterol and LDL cholesterol levels in plasma, with no significant change in high-density lipoprotein (HDL) [32]. Additionally, it can enhance lipid metabolism and insulin sensitivity through the SREBP-1c/PPARα signaling pathway, thereby preventing dietary obesity and related metabolic disorders [33]. The observed decrease in CHO and LDL levels in our study suggests that *L. acidophilus* may be a potential intervention for mitigating dietary obesity and related metabolic disorders.

The variable and stable regions of immunoglobulin interact synergistically to target the destruction and elimination of pathogenic microorganisms and toxins in the body [34]. IgA is widely recognized as a crucial antibody isotype responsible for safeguarding mucosal surfaces, its primary function involves immune exclusion, effectively preventing the entry of foreign substances [35]. Research has demonstrated that the unique C-terminal tail of IgA can impede the attachment of influenza A virus and other enveloped viruses using sialic acid as receptors on the cell surface [36]. Additionally, IgA can neutralize or clear pathogens by triggering mechanisms such as the IgA Fc receptor (FC-RI or CD89) on phagocytes, playing a crucial role in preventing the invasion of microorganisms on mucosal surfaces [37,38,39,40]. In our study, *L. acidophilus* significantly increased IgA serum content. This suggests that *L. acidophilus* can enhance the mucosal defense barrier in young dogs, effectively preventing the infection of specific pathogens.

Interferon regulatory factors (IRFs) constitute a family of transcription factors crucial in host defense, activating the transcription of IFN-α and other immune response genes upon activation. IFN-α occupies a pivotal role at the interface of innate and adaptive immunity in viral defense, making it instrumental in setting thresholds for autoimmunity. IFN-α enhances cytokine secretion, polyfunctionality, degranulation, and the cytotoxic potential of NK cells, while also augmenting viral inhibition by NK cells [41]. IL-2 serves as the principal growth factor for antigen-activated T lymphocytes, exerting control over autoimmunity through the production of CD4^+^ and CD25^+^ to regulate T cells [42]. In our study, both IFN-α and IL-2 serum levels increased to some extent, suggesting that *L. acidophilus* can enhance the body’s immunity. Regarding antioxidant effects, *L. acidophilus* ATCC 4356 has been shown to mitigate the development of atherosclerotic lesions in ApoE(−/−) mice by reducing oxidative stress and inflammatory responses [43]. The determination of antioxidant capacity helps to evaluate the physiological, environmental, and nutritional factors of the redox status of the body, which can provide information about the absorption and bioavailability of nutritional compounds [44]. Our results also support the notion that *L. acidophilus* possesses certain antioxidant capabilities.

The gastrointestinal tract serves as a pivotal physiological interface that integrates nutrient metabolism and microbiota-host interactions. During gut digestion, the collab-oration between hosts and microbes yields various enzymes, hormones, vitamins, and chemicals like short-chain fatty acids (SCFAs), bile acids, and conjugated linoleic acid (CLAs). These compounds play a critical role in regulating numerous host microbiome pathways, including those governing lipid levels. The antagonistic effect of *L. acidophilus* against pathogens and other organisms may stem from mechanisms such as nutrient and adhesion site competition, the production of metabolites like organic acids and hydrogen peroxide, as well as the synthesis of antibiotic-like compounds and bacteriocins [2]. Our study demonstrated that *L. acidophilus* significantly reduced the diversity of the fecal bacterial community and resulted in a more concentrated sample distribution in dogs, it may be that *L. acidophilus* inhibits the growth and reproduction of some bacterial communities. Dominant bacterial phyla in the LAC group included *Firmicutes*, *Bacteroidetes*, and *Fusobacteriota*, aligning with previous research findings [45,46]. Furthermore, the domestication of dogs was an important milestone of human civilization, a study of whole-genome resequencing in dogs and wolves showed that dogs had increased starch digestion function compared to wolves [47]. Our results showed that *Prevotella_9* and *Peptoclostridium* were dominant genera, and some specific bacteria were correlated with physiological indices (such as CHO, T-AOC, IgA, and IFN-α), suggesting that *L. acidophilus* might enhance lipid metabolism, immunity, and antioxidant performance by modulating the relative abundance of the bacterial community, which makes it easier to adapt to the urban lifestyle of low physical activity.

## 5. Conclusions

In our study, *L. acidophilus* plays an important role in improving lipid metabolism, immunity, and anti-oxidation by regulating specific bacterial communities, but the specific correlation and mechanism need to be verified by further research.

## Figures and Tables

**Table 1:** Composition and nutrient levels of basal diets (air-dry basis).

Items	Content %
Ingredients	
Corn	19.00
Soybean meal	8.00
Beef powder	4.00
Sweet potato granules	4.00
Broken rice	13.00
Beet pulp	8.00
Broken wall hemoglobulin powder	2.50
Fish meal	1.00
Bone meal	5.00
Chicken powder	10.00
Saccharomyces cerevisiae	5.00
Peameal	3.00
Chicken oil	11.00
Taste enhancer	1.70
Methionine	0.30
Chicken liver powder	2.50
Premix ^1^	2.00
Total	100.00
Nutrient levels ^2^	
CP	28.88
EE	8.85
Ash	6.24
Ca	0.89
P	0.66

^1^ Each kilogram of premix contained the following: VA 625,000 IU, VD3 100,000 IU, VE 6000 IU, VK3 200 mg, VB1 1250 mg, VB2 900 mg, VB6 750 mg, VB12 2.25 mg, biotin 10 mg, folic acid 150 mg, nicotinic acid 2500 mg, calcium pantothenate 1750 mg, VC 10,050 mg, choline 240,000 mg, Fe 9600 mg, Cu 18,000 mg, Zn 7800 mg, Mn 4800 mg, I 144 mg, Co 24 mg, Se 30 mg. CP: crude protein, EE: ether extract, Ash: crude ash. ^2^ Nutrient levels were measured values.

**Table 2:** Effects of *Lactobacillus acidophilus* on Body Weight in Chinese Rural Dogs.

Items	CON	LAC	*p*-Value
Body weight on day 7/kg	4.97 ± 0.52	5.23 ± 0.27	0.668
Body weight on day 14/kg	5.03 ± 0.53	5.38 ± 0.27	0.580
Body weight on day 21/kg	4.89 ± 0.61	5.22 ± 0.41	0.668
Body weight on day 28/kg	5.46 ± 0.57	5.73 ± 0.34	0.694
ADG (g/d)	30.95 ± 7.64	34.23 ± 6.85	0.756
WGR	0.20 ± 0.06	0.20 ± 0.04	0.984
TWG (kg)	0.87 ± 0.21	0.96 ± 0.19	0.756

In the same row, values with no letter superscripts represent no significant difference between groups (*p* > 0.05), The same as below. ADG: average daily weight gain, WGR: weight gain rate, TWG: total weight gain.

**Table 3:** Effect of *Lactobacillus acidophilus* on Apparent Digestibility of Nutrients in Chinese Rural Dogs.

Items	CON	LAC	*p*-Value
DM	77.69 ± 0.35	78.60 ± 1.42	0.559
CP	72.46 ± 0.96	72.24 ± 2.22	0.930
EE	95.95 ± 0.33	95.97 ± 0.30	0.965
Carbohydrates	83.31 ± 0.39	84.21 ± 1.17	0.491

DM: dry matter, CP: crude protein, EE: ether extract.

## Data Availability

The data that support the findings of this study are available from the corresponding author, [C.X.], upon reasonable request.
